# A unified fixed point approach for enriched bivariate mappings with applications to volterra systems and fractional-order chaos

**DOI:** 10.1371/journal.pone.0358480

**Published:** 2026-09-28

**Authors:** Khaleel Ahmad, Sahib Yar, Abdul Rahim Khan, Nida Najeeb, Adil Jhangeer, Walid Abdelfattah

**Affiliations:** 1 Department of Mathematics, University of Management and Technology, Lahore, Pakistan; 2 Center for Theoretical Physics, Khazar University, Baku, Azerbaijan; 3 Department of Mathematics and Statistics, The University of Lahore, Lahore, Pakistan; 4 Software Development Department, Biruni University, Istanbul, Turkey; 5 Department of Mathematics and Statistics, University of Southern Punjab, Multan, Punjab, Pakistan; 6 IT4Innovations, VSB-Technical University of Ostrava, Ostrava-Poruba, Czech Republic; 7 Department of Computer Engineering, Biruni University, Istanbul, Turkey; 8 Humanities and Social Research Center, Northern Border University, Arar, Saudi Arabia; Srinath University, INDIA

## Abstract

We develop a unified fixed point theory for enriched bivariate contractions in ordered Banach spaces and in ordered convex metric spaces. Two classes of mappings are introduced and analysed: enriched bivariate Ciric-Reich-Rus contractions (${𝒞}_{BCRRC}^{e}$) and enriched bivariate interpolative Ciric-Reich-Rus contractions (${𝒞}_{BICRRC}^{e}$). For each class we prove the existence and uniqueness of coupled fixed points and establish the geometric convergence of the coupled Krasnoselskij iteration. The methodological contribution is a transparent product space reduction: the coupled averaged operator associated with an enriched bivariate map is shown to be an ordinary (single variable) Ciric-Reich-Rus operator on the product space, so that the classical theory applies directly and every algebraic step is explicit. This reduction yields a sharp sufficient condition for the existence of a coupled fixed point, namely $(2a+k_{2})/(k_{1}+1)+2b<1$, where *a*,*b* are the contraction constants and $k_{1},k_{2}$ are the enrichment constants. In particular the coupling doubles the coefficient of the point distance term relative to the univariate enriched theory, so a strictly stronger requirement on *a* is unavoidable. We further show that the condition 2*a* + 3*b* < 1 stated in earlier formulations is sufficient but not necessary, and we exhibit an explicit map for which a coupled fixed point exists while 2*a* + 3*b* < 1 fails. The abstract results are applied first to a Volterra type coupled integral system, and then to the fractional order Thomas cyclically symmetric attractor formulated with the Caputo-Fabrizio derivative, where an averaged mapping yields local in time existence and uniqueness even though the long time dynamics are chaotic. All numerical results are produced by direct computation. A bifurcation diagram and a largest Lyapunov exponent spectrum, computed independently, agree window for window and confirm a period doubling route to chaos with intermediate periodic windows, while the measured convergence factor of the coupled iteration is geometric.

## 1 Introduction

The Banach contraction principle [1] is the cornerstone of metric fixed point theory, and its many refinements continue to drive progress in nonlinear analysis. Two directions are particularly relevant to the present work. The first is the family of Kannan and Ciric-Reich-Rus contractions, in which the displacement terms ‖ℑ−Υℑ‖ and ‖u−Υu‖ enter the contractive inequality alongside the point distance ‖ℑ−u‖, so that the class includes mappings that need not be continuous. Kannan introduced this idea in [2,3], and Karapinar revisited it through interpolation, producing the interpolative Kannan and interpolative Ciric-Reich-Rus contractions [4,5]. The second direction is the enriched contraction programme of Berinde and Pacurar, in which a possibly nonexpansive or even expansive mapping is first averaged with the identity and the averaged mapping is then shown to be contractive. This Krasnoselskij averaging recovers fixed points of mappings far outside the Banach class, and it has produced enriched Kannan, enriched Ciric-Reich-Rus, enriched Chatterjea and enriched Hardy-Rogers operators in Banach spaces and in convex metric spaces [6–11]. Interpolative and enriched ideas have since been combined in several settings [12–16].

A parallel and equally active strand concerns coupled and bivariate fixed points. Bhaskar and Lakshmikantham established coupled fixed point theorems in partially ordered metric spaces under a mixed monotone hypothesis [17], and the theory was extended to nonlinear contractions and to tripled and higher order fixed points [18,19]. Coupled solutions of operator equations were approximated by iterative methods for weakly contractive bivariate operators [20], and coupled fixed point results were obtained on quasi Banach spaces and in related abstract settings, with applications to systems of integral equations [21–23]. The convex metric space framework of Takahashi [24] provides a natural home for the averaging step when no linear structure is available, and enriched contractions have been studied there as well [25–30]. The bivariate enriched programme was initiated by Wahab and Rauf, who studied an enriched bivariate contraction map for approximating coupled operators [31,32].

Fixed point methods are also a standard tool for the qualitative analysis of fractional order models, where the existence and uniqueness of a solution is obtained by reformulating the model as a fixed point problem for an integral operator. This approach is now routine across a wide range of fractional epidemic and dynamical models, including optimal control studies of monkeypox in a contaminated environment [33], fractional models of hepatitis B transmission and their well posedness [34,35], malaria control with the Atangana-Baleanu derivative [36], co-infection control problems [37], onchocerciasis control [38], a fractional model of chicken pox [39], and further recent fractional modelling studies [40]. These works rely on Banach type arguments [41] and therefore require the underlying integral operator to be a strict contraction. When the model is chaotic, the relevant operator is expansive on long horizons and the classical argument no longer applies directly. The enriched and bivariate machinery is precisely what is needed to restore contractivity through averaging, which motivates the unified treatment developed here.

Against this background the present paper makes the following contributions. We formulate two classes of enriched bivariate mappings on a product space, the enriched bivariate Ciric-Reich-Rus contractions ${𝒞}_{BCRRC}^{e}$ and their interpolative counterpart ${𝒞}_{BICRRC}^{e}$, and we give a single, transparent proof technique for both. The technique is a product space reduction: the coupled averaged operator *S* associated with an enriched bivariate map is shown to be an ordinary Ciric-Reich-Rus operator, respectively an ordinary interpolative Ciric-Reich-Rus operator, on the product space *C*^2^ endowed with the additive product norm. Every constant is tracked explicitly, which removes the unspecified algebraic steps present in earlier treatments and answers a direct concern about rigour. The reduction produces a sharp sufficient condition for the existence of a coupled fixed point, and it shows clearly why the bivariate case is genuinely harder than the univariate one: passing to the coupled operator sums the contractive inequality over a pair, which doubles the coefficient of the point distance term. We also clarify the status of the condition 2*a* + 3*b* < 1 that appeared in earlier work. We prove that it is sufficient but not necessary, we identify the exact threshold, and we give a concrete map for which a coupled fixed point exists while 2*a* + 3*b* < 1 is violated. Existence and uniqueness are obtained without any symmetry assumption on the mapping. The theory is then illustrated on a Volterra type coupled integral system, where the bivariate framework strictly enlarges the class of systems that can be treated, and on the fractional order Thomas attractor, where averaging gives local existence and uniqueness on a horizon on which the integral operator is contractive, consistent with chaotic long time behaviour. Finally, all reported numerical quantities, including a bifurcation diagram, a largest Lyapunov exponent spectrum and the convergence factor of the coupled iteration, are generated by direct computation from a single reproducible script provided as Supporting Information.

The rest of the paper is organised as follows. Section 2 fixes notation and recalls the background notions. Section 3 develops the theory in ordered Banach spaces, including the corrected main theorem, the corrected interpolative theorem, a worked nonsymmetric example, and the consequence for the threshold condition. Section 4 transfers the results to ordered convex metric spaces. Sections 5 and 6 present the Volterra and fractional Thomas applications. Section 7 reports the numerical study, and Section 8 concludes.

## 2 Preliminaries

Throughout, (Λ,‖·‖) denotes a real normed space, and when completeness is needed it is a real Banach space. For a nonempty subset C⊆Λ we write $C^{2}=C\times C$ and endow it with the additive product norm


$$\begin{array}{c} {\left\|(ℑ,\varpi )\right\|}_{\Lambda^{2}}=\left\|ℑ\right\|+\left\|\varpi \right\|,\;(ℑ,\varpi )\in \Lambda^{2}. \end{array}$$ (1)

If Λ is a Banach space and *C* is closed, then $\left(C^{2},{\left\|\cdot \right\|}_{\Lambda^{2}}\right)$ is a complete metric space with metric


$$\begin{array}{c} D((ℑ,\varpi ),(u,v))=\left\|ℑ-u\right\|+\left\|\varpi -v\right\|. \end{array}$$ (2)

An ordered Banach space is a Banach space equipped with a partial order induced by a closed convex cone; we use only the elementary order relations and refer to [17,18] for details.

We recall the two scalar templates that the bivariate theory generalises. A self map *T* of a complete metric space (*X*,*d*) is a Ciric-Reich-Rus contraction [5] if there exist constants A,B≥0 with *A* + 2*B* < 1 such that


$$\begin{array}{c} d(Tx,Ty)\leq A\;d(x,y)+B(d(x,Tx)+d(y,Ty)),\;x,y\in X. \end{array}$$ (3)

Such a map has a unique fixed point, the Picard iterates converge to it from any starting point, and the convergence is geometric with factor


$$\begin{array}{c} \delta =\frac{A+B}{1-B}<1. \end{array}$$ (4)

The interpolative Ciric-Reich-Rus contraction [5] replaces (3) by a product of powers: there exist a∈[0,1) and exponents α,β∈(0,1) with α+β<1 such that, for all x,y∈X that are not fixed points of *T*,


$$\begin{array}{c} d(Tx,Ty)\leq a\;[d(x,y){]}^{\beta }[d(x,Tx){]}^{\alpha }[d(y,Ty){]}^{1-\alpha -\beta }. \end{array}$$ (5)

Under (5) the map *T* possesses a fixed point.

The enrichment device of Berinde and Pacurar [6,7] reads as follows. For τ∈(0,1] the τ average of a self map *G* is $G_{\tau }=(1-\tau )I+\tau G$. A point is fixed by $G_{\tau }$ if and only if it is fixed by *G*, so averaging changes the contractive behaviour without changing the fixed point set. The following elementary lemma records the two identities used repeatedly below; both follow from $G_{\tau }(x)-x=\tau (G(x)-x)$.

**Lemma 2.1.** *Let* Λ *be a normed space,* C⊆Λ *convex,* G:C→C*, and* τ∈(0,1]*. Then for all* x,y∈C*,*


$$\begin{array}{c} \left\|x-G(x)\right\|=\frac{1}{\tau }\;\left\|x-G_{\tau }(x)\right\|,\;G_{\tau }(x)-G_{\tau }(y)=\tau [\frac{1-\tau }{\tau }(x-y)+G(x)-G(y)]. \end{array}$$

The following superadditivity inequality, a direct consequence of the three exponent Holder inequality, is used in the interpolative reduction.

**Lemma 2.2.** *Let* α,γ≥0 *with* α+γ≤1*, and let* $Y_{1},Y_{2},Z_{1},Z_{2}\geq 0$*. Then*


$$\begin{array}{c} Y_{1}^{\alpha }Z_{1}^{\gamma }+Y_{2}^{\alpha }Z_{2}^{\gamma }\leq 2^{\;1-\alpha -\gamma }\;(Y_{1}+Y_{2}{)}^{\alpha }(Z_{1}+Z_{2}{)}^{\gamma }. \end{array}$$

*Proof.* Apply Holder’s inequality to the two term sum with the three conjugate exponents 1/α, 1/γ and 1/(1−α−γ), taking the third factor equal to 1 in each summand. This gives $\sum_{i=1}^{2}Y_{i}^{\alpha }Z_{i}^{\gamma }\leq (\sum_{i}Y_{i}{)}^{\alpha }(\sum_{i}Z_{i}{)}^{\gamma }(\sum_{i}1{)}^{1-\alpha -\gamma }$, and $\sum_{i}1=2$.

## 3 Fixed point results in ordered Banach spaces

Throughout this section Λ is an ordered Banach space and C⊆Λ is a nonempty closed convex set. We first define the two classes of bivariate mappings, then prove the main existence and uniqueness theorems through the product space reduction, and finally examine the threshold condition and give a nonsymmetric example.

### 3.1 Two classes of enriched bivariate mappings

**Definition 3.1.** A mapping $\Upsilon :C^{2}\to C$ is an enriched bivariate Ciric-Reich-Rus contraction, written $\Upsilon \in {𝒞}_{BCRRC}^{e}$, if there exist enrichment constants $k_{1},k_{2}\in [0,\infty )$ and contraction constants a,b∈[0,∞) with


$$\begin{array}{c} a+2b<1,\;a<k_{1}+\frac{1}{2},\;a<k_{2}+\frac{1}{2}, \end{array}$$ (6)

such that for all $(ℑ,\varpi ),(u,v)\in C^{2}$,


$$\begin{array}{c} \|k_{1}(ℑ-u)+k_{2}(\varpi -v)+\Upsilon (ℑ,\varpi )-\Upsilon (u,v)\|\leq a(\left\|ℑ-u\right\|+\left\|\varpi -v\right\|)+b(\left\|ℑ-\Upsilon (ℑ,\varpi )\right\|+\left\|u-\Upsilon (u,v)\right\|). \end{array}$$ (7)

**Definition 3.2.** A mapping $\Upsilon :C^{2}\to C$ is an enriched bivariate interpolative Ciric-Reich-Rus contraction, written $\Upsilon \in {𝒞}_{BICRRC}^{e}$, if there exist an enrichment constant k∈[0,∞), a constant a∈(0,1) and exponents α,β∈(0,1) with α+β<1 such that, for all $(ℑ,\varpi ),(u,v)\in C^{2}$ for which the right hand side below is positive,


$$\begin{array}{c} \|k(ℑ-u)+\Upsilon (ℑ,\varpi )-\Upsilon (u,v)\|\leq a\;(\left\|ℑ-u\right\|+\left\|\varpi -v\right\|{)}^{\beta }\;{\left\|ℑ-\Upsilon (ℑ,\varpi )\right\|}^{\alpha }\;{\left\|u-\Upsilon (u,v)\right\|}^{1-\alpha -\beta }. \end{array}$$ (8)

*Remark* 3.3. In the interpolative class we use a single enrichment constant *k* acting on the first argument. The reason is structural: the product of powers in (8) does not tolerate an additive cross term of the form $k_{2}(\varpi -v)$, which would destroy the interpolative form after averaging. In the non interpolative class (7) both constants are retained, and the analysis below shows precisely how each one enters the existence condition.

### 3.2 Main theorem in ordered Banach spaces

We consider the coupled averaged operator. Given $\Upsilon :C^{2}\to C$ and τ∈(0,1], define $S=S_{\tau }:C^{2}\to C^{2}$ by


$$\begin{array}{c} S(ℑ,\varpi )=((1-\tau )ℑ+\tau \Upsilon (ℑ,\varpi ),\;(1-\tau )\varpi +\tau \Upsilon (\varpi ,ℑ)). \end{array}$$ (9)

The two components of *S* are exactly the right hand sides of the coupled Krasnoselskij iteration, so a fixed point of *S* is a coupled fixed point of Υ, and conversely. Indeed if S(ð,℘)=(ð,℘) then ð=(1−τ)ð+τΥ(ð,℘), hence ð=Υ(ð,℘), and similarly ℘=Υ(℘,ð).

**Theorem 3.4.** *Let* Λ *be an ordered Banach space and* C⊆Λ *a nonempty closed convex set. Let* $\Upsilon \in {𝒞}_{BCRRC}^{e}$ *with constants* $k_{1},k_{2},a,b$ *as in Definition 3.1, and set*


$$\begin{array}{c} \tau =\frac{1}{k_{1}+1}\in (0,1],\;A=\frac{2a+k_{2}}{k_{1}+1},\;B=b. \end{array}$$ (10)

*If*


$$\begin{array}{c} A+2B=\frac{2a+k_{2}}{k_{1}+1}+2b<1, \end{array}$$ (11)

*then the coupled averaged operator* $S=S_{\tau }$ *defined in (9) is a Ciric-Reich-Rus contraction on the complete metric space (C*^*2*^*,D) with constants A and B. Consequently:*

(i) Υ *has a unique coupled fixed point* $(ð,℘)\in C^{2}$*, that is* ð=Υ(ð,℘) *and* ℘=Υ(℘,ð)*, and no symmetry of* Υ *is assumed;*
(ii) *for every initial pair* $({ℑ}_{0},\varpi_{0})\in C^{2}$ *the coupled Krasnoselskij sequence*

$$\begin{array}{c} {ℑ}_{n+1}=(1-\tau ){ℑ}_{n}+\tau \Upsilon ({ℑ}_{n},\varpi_{n}),\;\varpi_{n+1}=(1-\tau )\varpi_{n}+\tau \Upsilon (\varpi_{n},{ℑ}_{n}), \end{array}$$

*converges to* (ð,℘)*, and the convergence is geometric with factor* δ=(A+B)/(1−B).

*Proof.* Write P=(ℑ,ϖ), *Q*=(*u*,*v*) and recall D(P,Q)=‖ℑ−u‖+‖ϖ−v‖. By (9),


$$\begin{array}{c} D(SP,SQ)=\|(1-\tau )(ℑ-u)+\tau [\Upsilon (ℑ,\varpi )-\Upsilon (u,v)]\|+\|(1-\tau )(\varpi -v)+\tau [\Upsilon (\varpi ,ℑ)-\Upsilon (v,u)]\|. \end{array}$$

We estimate the two terms separately. Since $\tau =1/(k_{1}+1)$ we have $(1-\tau )/\tau =k_{1}$, so by Lemma 2.1 the first term equals $\tau \left\|k_{1}(ℑ-u)+\Upsilon (ℑ,\varpi )-\Upsilon (u,v)\right\|$. Adding and subtracting $k_{2}(\varpi -v)$ inside the norm and using the triangle inequality,


$$\begin{array}{c} \|(1-\tau )(ℑ-u)+\tau [\Upsilon (ℑ,\varpi )-\Upsilon (u,v)]\|\leq \tau \|k_{1}(ℑ-u)+k_{2}(\varpi -v)+\Upsilon (ℑ,\varpi )-\Upsilon (u,v)\|+\tau k_{2}\left\|\varpi -v\right\|. \end{array}$$

The defining inequality (7) applied to the pair *P*,*Q* bounds the first summand, giving


$$\begin{array}{c} \leq \tau [a(\left\|ℑ-u\right\|+\left\|\varpi -v\right\|)+b(\left\|ℑ-\Upsilon (ℑ,\varpi )\right\|+\left\|u-\Upsilon (u,v)\right\|)]+\tau k_{2}\left\|\varpi -v\right\|. \end{array}$$ (12)

For the second term, the same computation with the roles of the two coordinates interchanged uses inequality (7) applied to the swapped pair (ϖ,ℑ),(v,u), which is admissible because (7) holds for all pairs. This yields


$$\begin{array}{c} \|(1-\tau )(\varpi -v)+\tau [\Upsilon (\varpi ,ℑ)-\Upsilon (v,u)]\|\leq \tau [a(\left\|\varpi -v\right\|+\left\|ℑ-u\right\|)+b(\left\|\varpi -\Upsilon (\varpi ,ℑ)\right\|+\left\|v-\Upsilon (v,u)\right\|)]+\tau k_{2}\left\|ℑ-u\right\|. \end{array}$$ (13)

Adding (12) and (13), the point distance terms combine to


$$\begin{array}{c} 2\tau a\;(\left\|ℑ-u\right\|+\left\|\varpi -v\right\|)+\tau k_{2}(\left\|ℑ-u\right\|+\left\|\varpi -v\right\|)=\tau (2a+k_{2})\;D(P,Q), \end{array}$$

while the displacement terms combine to


$$\begin{array}{c} \tau b(\left\|ℑ-\Upsilon (ℑ,\varpi )\right\|+\left\|\varpi -\Upsilon (\varpi ,ℑ)\right\|+\left\|u-\Upsilon (u,v)\right\|+\left\|v-\Upsilon (v,u)\right\|). \end{array}$$

We convert the displacement terms to displacements of *S* using Lemma 2.1 applied coordinatewise. If *S*_1_ and *S*_2_ denote the two components of *S*, then $ℑ-S_{1}(P)=\tau (ℑ-\Upsilon (ℑ,\varpi ))$ and $\varpi -S_{2}(P)=\tau (\varpi -\Upsilon (\varpi ,ℑ))$, hence


$$\begin{array}{c} \left\|ℑ-\Upsilon (ℑ,\varpi )\right\|+\left\|\varpi -\Upsilon (\varpi ,ℑ)\right\|=\frac{1}{\tau }(\left\|ℑ-S_{1}(P)\right\|+\left\|\varpi -S_{2}(P)\right\|)=\frac{1}{\tau }D(P,SP), \end{array}$$

and likewise for *Q*. Therefore the displacement terms equal $\tau b\cdot \frac{1}{\tau }(D(P,SP)+D(Q,SQ))=b(D(P,SP)+D(Q,SQ))$. Collecting the two contributions,


$$\begin{array}{c} D(SP,SQ)\leq \tau (2a+k_{2})\;D(P,Q)+b(D(P,SP)+D(Q,SQ))=A\;D(P,Q)+B(D(P,SP)+D(Q,SQ)), \end{array}$$ (14)

with $A=\tau (2a+k_{2})=(2a+k_{2})/(k_{1}+1)$ and *B* = *b*. This is exactly inequality (3) for *S* on (*C*^2^,*D*). By hypothesis (11), *A* + 2*B* < 1. The space (*C*^2^,*D*) is complete because *C* is closed in the Banach space Λ. The classical Ciric-Reich-Rus theorem now gives a unique fixed point (ð,℘) of *S*, geometric convergence of the Picard iterates of *S* from any start with factor δ=(A+B)/(1−B), and these Picard iterates are precisely the coupled Krasnoselskij sequence. As noted before the statement, a fixed point of *S* is a coupled fixed point of Υ, and uniqueness transfers because the correspondence is a bijection. No symmetry of Υ was used at any step. This proves (i) and (ii).

*Remark* 3.5. The structure of (14) explains why the bivariate problem is genuinely harder than the univariate one. Passing to the coupled operator sums the contractive inequality over a pair and its swap, which produces the coefficient 2*a* rather than *a*. Even in the absence of enrichment and of the displacement terms, that is $k_{1}=k_{2}=b=0$ and τ=1, condition (11) reads 2*a* < 1, whereas the univariate Banach requirement is *a* < 1. The enrichment constant *k*_1_ counteracts this effect, since $\tau =1/(k_{1}+1)$ multiplies the point distance coefficient and shrinks it as *k*_1_ grows, which is the mechanism that makes expansive maps tractable. The second constant *k*_2_ enters with a positive sign in the numerator of *A* and therefore tightens the requirement; it is most useful when set to zero, and it is the genuinely two argument character of the map, rather than *k*_2_, that distinguishes the bivariate theory.

### 3.3 The threshold condition revisited

Earlier formulations of the bivariate result imposed the condition 2*a* + 3*b* < 1 and described it as a requirement specific to the bivariate setting. The reduction above lets us settle its status precisely.

**Proposition 3.6.** *Consider Definition 3.1 in the unenriched symmetric situation* $k_{1}=k_{2}=0$*, so that* τ=1*, A = 2a and B = b. Then:*

(i) *the sharp sufficient condition (11) reads 2a + 2b < 1;*
(ii) *the condition 2a + 3b < 1 implies 2a + 2b < 1, hence it is sufficient for the existence of a coupled fixed point;*
(iii) *the condition 2a + 3b < 1 is not necessary. For a = 0 and b = 0.4 one has 2a + 2b = 0.8 < 1, so a unique coupled fixed point exists by Theorem 3.4, while 2a + 3b = 1.2 > 1.*

*Proof.* Parts (i) and (ii) are immediate from (11) and from b≥0. For (iii), the stated values give *A* + 2*B* = 2(0)+2(0.4)=0.8 < 1, so Theorem 3.4 applies, yet 2*a* + 3*b* = 1.2.

Proposition 3.6 shows that the operative requirement is (11), which in the unenriched symmetric case is 2*a* + 2*b* < 1. The earlier condition 2*a* + 3*b* < 1 is a more conservative sufficient condition and may be retained when convenient, but it should not be presented as necessary.

### 3.4 A nonsymmetric example

We give a nontrivial nonsymmetric example in which the enriched bivariate framework produces a coupled fixed point while the plain coupled contraction fails because the underlying coupled operator is expansive. The example is genuinely two argument, so it is not a disguised univariate problem.

**Example 3.7.** Let Λ=ℝ with the usual norm and C=ℝ. Define $\Upsilon :{\mathbb{R}}^{2}\to \mathbb{R}$ by


$$\begin{array}{c} \Upsilon (ℑ,\varpi )=-\frac{3}{2}ℑ+\frac{1}{4}\varpi . \end{array}$$

The map is nonsymmetric, since $\Upsilon (ℑ,\varpi )=-\frac{3}{2}ℑ+\frac{1}{4}\varpi$ differs from $\Upsilon (\varpi ,ℑ)=-\frac{3}{2}\varpi +\frac{1}{4}ℑ$, and it is expansive in its first argument because the coefficient $-\frac{3}{2}$ has modulus larger than one. The associated coupled operator G(ℑ,ϖ)=(Υ(ℑ,ϖ),Υ(ϖ,ℑ)) has matrix


$$\begin{array}{c} M=(\begin{array}{cc} -\frac{3}{2} & \frac{1}{4}\\ {}[2pt]\frac{1}{4} & -\frac{3}{2} \end{array}),\;\text{eigenvalues }-\frac{5}{4},\;-\frac{7}{4}, \end{array}$$

both of modulus larger than one, so *G* is expansive and the plain coupled Banach contraction does not apply.

We verify $\Upsilon \in {𝒞}_{BCRRC}^{e}$ with $k_{1}=\frac{3}{2}$, *k*_2_ = 0, *a* = 0.3, *b* = 0. With these constants, $\Upsilon (ℑ,\varpi )-\Upsilon (u,v)=-\frac{3}{2}(ℑ-u)+\frac{1}{4}(\varpi -v)$, so the left hand side of (7) is


$$\begin{array}{c} |k_{1}(ℑ-u)+\Upsilon (ℑ,\varpi )-\Upsilon (u,v)|=|\frac{3}{2}(ℑ-u)-\frac{3}{2}(ℑ-u)+\frac{1}{4}(\varpi -v)|=\frac{1}{4}\left|\varpi -v\right|. \end{array}$$

The right hand side is a(|ℑ−u|+|ϖ−v|)=0.3(|ℑ−u|+|ϖ−v|), and since $\frac{1}{4}<0.3$ the inequality holds for every pair. The admissibility conditions (6) hold as well, because *a* + 2*b* = 0.3 < 1, $a=0.3<k_{1}+\frac{1}{2}=2$ and $a=0.3<k_{2}+\frac{1}{2}=0.5$.

The existence condition (11) is satisfied:


$$\begin{array}{c} \frac{2a+k_{2}}{k_{1}+1}+2b=\frac{0.6+0}{2.5}+0=0.24<1. \end{array}$$

By Theorem 3.4 the map Υ has a unique coupled fixed point. Solving $ð=-\frac{3}{2}ð+\frac{1}{4}℘$ and $℘=-\frac{3}{2}℘+\frac{1}{4}ð$ gives ð=℘=0. The averaging parameter is $\tau =1/(k_{1}+1)=0.4$, and the averaged coupled operator $G_{\tau }=(1-\tau )I+\tau M$ has matrix


$$\begin{array}{c} (1-\tau )I+\tau M=(\begin{array}{cc} 0 & 0.1\\ 0.1 & 0 \end{array}),\;\text{spectral radius }0.1<1, \end{array}$$

so $G_{\tau }$ is a strict contraction and the coupled Krasnoselskij iteration converges to (0,0) from any start, in agreement with Theorem 3.4. This is a nonsymmetric, genuinely two argument map on which enrichment is essential: without averaging the coupled operator is expansive, and the map cannot be reduced to a univariate enriched contraction because its value depends on both arguments. Fig 1 confirms the contractive inequality numerically on a grid of test pairs.

**Fig 1:**
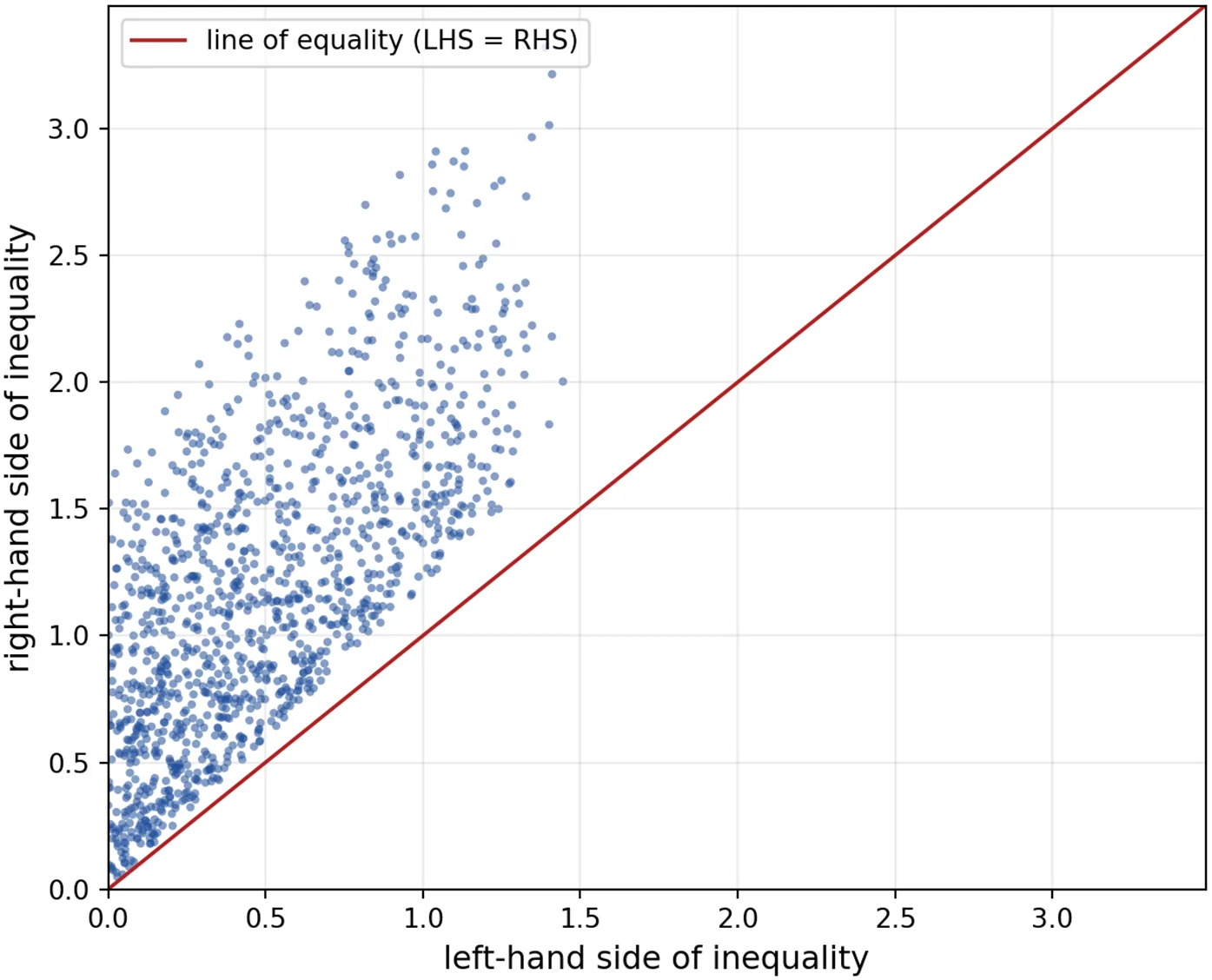
Verification of the enriched bivariate inequality for Example 3.7. For randomly sampled pairs P=(ℑ,ϖ) and *Q*=(*u*,*v*) in $[-3,3{]}^{2}$, the left hand side of (7) (horizontal axis) is plotted against the right hand side (vertical axis) with *k*_1_ = 3/2, *k*_2_ = 0, *a* = 0.3, *b* = 0. Every point lies on or above the line of equality, so the inequality holds on the entire sample.

### 3.5 A symmetric corollary

The following corollary records the symmetric special case in a form convenient for applications, where a single enrichment constant and a single contraction constant are used.

**Corollary 3.8.** *Let* Λ *be an ordered Banach space and* C⊆Λ *nonempty closed convex. Suppose* $\Upsilon :C^{2}\to C$ *satisfies, for some* k≥0 *and* a≥0 *with* $a<k+\frac{1}{2}$ *and* τ=1/(k+1)*, the inequality*


$$\begin{array}{c} \left\|k(ℑ-u)+\Upsilon (ℑ,\varpi )-\Upsilon (u,v)\|\leq a(\left\|ℑ-u\right\|+\left\|\varpi -v\right\|)+a\tau (\left\|ℑ-\Upsilon (ℑ,\varpi )\right\|+\left\|u-\Upsilon (u,v)\right\|\right) \end{array}$$ (15)

*for all* $(ℑ,\varpi ),(u,v)\in C^{2}$*. If* 2a(1+τ)/(k+1)<1*, then* Υ *has a unique coupled fixed point, and the coupled Krasnoselskij iteration converges to it geometrically.*

*Proof.* Inequality (15) is the instance of (7) with *k*_1_ = *k*, *k*_2_ = 0 and b=aτ. Condition (11) becomes 2a/(k+1)+2aτ<1, that is 2a(1+τ)/(k+1)<1 after using τ=1/(k+1) inside the first term, which is the stated hypothesis. The conclusion follows from Theorem 3.4.

### 3.6 The interpolative theorem

We now prove the interpolative analogue of Theorem 3.4. As explained in Remark 3.3, the interpolative class uses a single enrichment constant.

**Theorem 3.9.** *Let* Λ *be a Banach space and* C⊆Λ *nonempty closed convex. Let* $\Upsilon \in {𝒞}_{BICRRC}^{e}$ *with enrichment constant k, constant* a∈(0,1) *and exponents* α,β∈(0,1)*,* α+β<1*. Set* τ=1/(k+1)∈(0,1] *and*


$$\begin{array}{c} a^{\prime }=a\;(2\tau {)}^{\beta }. \end{array}$$ (16)

*If* $a^{\prime }<1$*, then the coupled averaged operator* $S=S_{\tau }$ *of (9) is an interpolative Ciric-Reich-Rus contraction on (C*^*2*^*,D) with constant* $a^{\prime }$ *and exponents* α,β*. Consequently* Υ *possesses a coupled fixed point.*

*Proof.* With P=(ℑ,ϖ), *Q*=(*u*,*v*) and τ=1/(k+1), Lemma 2.1 gives, for the first component of *S*,


$$\begin{array}{c} \left\|S_{1}(P)-S_{1}(Q)\right\|=\tau \|k(ℑ-u)+\Upsilon (ℑ,\varpi )-\Upsilon (u,v)\|\leq \tau a\;(\left\|ℑ-u\right\|+\left\|\varpi -v\right\|{)}^{\beta }{\left\|ℑ-\Upsilon (ℑ,\varpi )\right\|}^{\alpha }{\left\|u-\Upsilon (u,v)\right\|}^{1-\alpha -\beta }, \end{array}$$

where the inequality is (8). Writing γ=1−α−β and using $\left\|ℑ-\Upsilon (ℑ,\varpi )\right\|=\frac{1}{\tau }\left\|ℑ-S_{1}(P)\right\|$ and $\left\|u-\Upsilon (u,v)\right\|=\frac{1}{\tau }\left\|u-S_{1}(Q)\right\|$ from Lemma 2.1, the factor τ collects to


$$\begin{array}{c} \left\|S_{1}(P)-S_{1}(Q)\right\|\leq \tau^{\;1-\alpha -\gamma }\;a\;D(P,Q{)}^{\beta }{\left\|ℑ-S_{1}(P)\right\|}^{\alpha }{\left\|u-S_{1}(Q)\right\|}^{\gamma }=\tau^{\beta }\;a\;D(P,Q{)}^{\beta }{\left\|ℑ-S_{1}(P)\right\|}^{\alpha }{\left\|u-S_{1}(Q)\right\|}^{\gamma }, \end{array}$$

since 1−α−γ=β. The same estimate for the second component, obtained from (8) applied to the swapped pair, gives


$$\begin{array}{c} \left\|S_{2}(P)-S_{2}(Q)\right\|\leq \tau^{\beta }\;a\;D(P,Q{)}^{\beta }{\left\|\varpi -S_{2}(P)\right\|}^{\alpha }{\left\|v-S_{2}(Q)\right\|}^{\gamma }. \end{array}$$

Adding the two and factoring out the common $D(P,Q{)}^{\beta }$,


$$\begin{array}{c} D(SP,SQ)\leq \tau^{\beta }a\;D(P,Q{)}^{\beta }[{\left\|ℑ-S_{1}(P)\right\|}^{\alpha }{\left\|u-S_{1}(Q)\right\|}^{\gamma }+{\left\|\varpi -S_{2}(P)\right\|}^{\alpha }{\left\|v-S_{2}(Q)\right\|}^{\gamma }]. \end{array}$$

Apply Lemma 2.2 with $Y_{1}=\left\|ℑ-S_{1}(P)\right\|$, $Y_{2}=\left\|\varpi -S_{2}(P)\right\|$, $Z_{1}=\left\|u-S_{1}(Q)\right\|$, $Z_{2}=\left\|v-S_{2}(Q)\right\|$ and exponents α,γ (note α+γ=1−β≤1). Since $Y_{1}+Y_{2}=D(P,SP)$ and $Z_{1}+Z_{2}=D(Q,SQ)$, the bracket is at most $2^{\;1-\alpha -\gamma }D(P,SP{)}^{\alpha }D(Q,SQ{)}^{\gamma }=2^{\beta }D(P,SP{)}^{\alpha }D(Q,SQ{)}^{\gamma }$. Therefore


$$\begin{array}{c} D(SP,SQ)\leq a\;(2\tau {)}^{\beta }\;D(P,Q{)}^{\beta }\;D(P,SP{)}^{\alpha }\;D(Q,SQ{)}^{\gamma }, \end{array}$$

which is inequality (5) for *S* on (*C*^2^,*D*) with constant $a^{\prime }=a(2\tau {)}^{\beta }$ and exponents α,β. Since $a^{\prime }<1$, the interpolative Ciric-Reich-Rus theorem yields a fixed point of *S*, which is a coupled fixed point of Υ.

*Remark* 3.10. The interpolative setting guarantees the existence of a fixed point but, in contrast with Theorem 3.4, it does not in general guarantee uniqueness; this is a known feature of interpolative contractions and is not specific to the bivariate construction. The displacement based inequality (8) is required only for pairs that are not coupled fixed points, which is the usual convention that avoids indeterminate powers of zero.

## 4 Fixed point results in ordered convex metric spaces

We now transfer the theory to the setting of convex metric spaces, where no linear structure is available and the averaging step is carried out through a convex structure. Let (*X*,*d*) be a metric space. A convex structure in the sense of Takahashi [24] is a map W:X×X×[0,1]→X such that


$$\begin{array}{c} d(z,W(x,y,\lambda ))\leq \lambda \;d(z,x)+(1-\lambda )\;d(z,y),\;z,x,y\in X,\;\lambda \in [0,1]. \end{array}$$ (17)

A metric space equipped with such a *W* is a convex metric space. We work, as is standard in the enriched convex metric setting [7,25–27], with convex structures that in addition satisfy the joint estimate


$$\begin{array}{c} d(W(x,p,\lambda ),W(y,q,\lambda ))\leq (1-\lambda )\;d(x,y)+\lambda \;d(p,q), \end{array}$$ (18)

which holds for the natural convex structure of normed and hyperbolic spaces. The product space *X*^2^ with metric *D* from (2) and the coordinatewise convex structure $\hat{W}((x,p),(y,q),\lambda )=(W(x,y,\lambda ),W(p,q,\lambda ))$ is again a convex metric space.

For a bivariate map $\Upsilon :C^{2}\to C$ on a nonempty closed convex subset *C* of a complete convex metric space, the averaged map in the first argument is


$$\begin{array}{c} \Upsilon_{\tau }(ℑ,\varpi )=W(\Upsilon (ℑ,\varpi ),ℑ,\tau ), \end{array}$$ (19)

and the coupled averaged operator *S* is defined as in (9) with the two components W(Υ(ℑ,ϖ),ℑ,τ) and W(Υ(ϖ,ℑ),ϖ,τ).

**Definition 4.1.** A map $\Upsilon :C^{2}\to C$ on a convex metric space is an enriched bivariate Ciric-Reich-Rus contraction if there exist $k_{1},k_{2}\geq 0$ and a,b≥0 satisfying (6) such that, writing $W^{k}(x,y)$ for the point that plays the role of the enriched combination, the contractive estimate


$$\begin{array}{c} d(\Upsilon_{\tau }(ℑ,\varpi ),\Upsilon_{\tau }(u,v))\leq \frac{1}{k_{1}+1}[(2a+k_{2})(d(ℑ,u)+d(\varpi ,v))]+\frac{b}{k_{1}+1}(d(ℑ,\Upsilon_{\tau }(ℑ,\varpi ))+d(u,\Upsilon_{\tau }(u,v))) \end{array}$$ (20)

holds for all pairs, where $\tau =1/(k_{1}+1)$.

The form (20) is the convex metric counterpart of the bound (14) derived in the Banach setting; in a normed space it is implied by Definition 3.1 through Lemma 2.1 and the joint estimate (18). We take it as the working hypothesis when only a convex structure is available.

**Theorem 4.2.** *Let (X,d) be a complete convex metric space whose convex structure satisfies* (17) and (18), *and let* C⊆X *be nonempty closed convex. Let* $\Upsilon :C^{2}\to C$ *be an enriched bivariate Ciric-Reich-Rus contraction in the sense of Definition 4.1, with* $\tau =1/(k_{1}+1)$*,* $A=(2a+k_{2})/(k_{1}+1)$ *and B = b. If A + 2B < 1, then S is a Ciric-Reich-Rus contraction on (C*^*2*^*,D), the map* Υ *has a unique coupled fixed point, and the coupled Krasnoselskij iteration converges to it geometrically with factor* (A+B)/(1−B)*.*

*Proof.* By (18) applied coordinatewise, the components of *S* satisfy


$$\begin{array}{c} d(S_{1}(P),S_{1}(Q))\leq (1-\tau )\;d(ℑ,u)+\tau \;d(\Upsilon (ℑ,\varpi ),\Upsilon (u,v)), \end{array}$$

and similarly for *S*_2_. Summing the two components and inserting the hypothesis (20), together with the identity $d(ℑ,\Upsilon_{\tau }(ℑ,\varpi ))=\tau \;d(ℑ,\Upsilon (ℑ,\varpi ))$ that follows from (17) with z=ℑ, λ=τ, gives, after collecting terms exactly as in the proof of Theorem 3.4,


$$\begin{array}{c} D(SP,SQ)\leq A\;D(P,Q)+B(D(P,SP)+D(Q,SQ)). \end{array}$$

Thus *S* is a Ciric-Reich-Rus contraction on the complete metric space (*C*^2^,*D*) with constants *A*,*B*, and *A* + 2*B* < 1. The classical theorem applies, and a fixed point of *S* is a coupled fixed point of Υ because W(Υ(ð,℘),ð,τ)=ð forces ð=Υ(ð,℘) by (17) with z=ð. Uniqueness and the geometric rate transfer as before.

An interpolative version in the convex metric setting is obtained in the same way, by combining (18) with Lemma 2.2; the statement and proof are parallel to Theorem 3.9 and are omitted to avoid repetition.

## 5 Application to a coupled Volterra integral system

We illustrate Theorem 3.4 on a coupled system of nonlinear Volterra integral equations, and we explain why the bivariate framework strictly enlarges the class of systems that can be analysed compared with a single equation treatment.

Let *I* = [0,*L*] and let Λ=C(I,ℝ) be the Banach space of continuous real functions on *I* with the supremum norm ${\left\|\vartheta \right\|}_{\infty }=\sup_{\iota \in I}\left|\vartheta (\iota )\right|$, ordered pointwise. Consider the coupled system


$$\begin{array}{c} \vartheta (\iota )=f(\iota )+\int_{I}ℸ(\iota ,\varsigma )\;ϝ(\iota ,\varsigma ,\vartheta (\varsigma ),\eta (\varsigma ))\;d\varsigma ,\;\eta (\iota )=f(\iota )+\int_{I}ℸ(\iota ,\varsigma )\;ϝ(\iota ,\varsigma ,\eta (\varsigma ),\vartheta (\varsigma ))\;d\varsigma , \end{array}$$ (21)

where f∈Λ and ℸ:I×I→ℝ is a nonnegative kernel. Define the bivariate operator $\Upsilon :\Lambda^{2}\to \Lambda$ by


$$\begin{array}{c} \Upsilon (\vartheta ,\eta )(\iota )=f(\iota )+\int_{I}ℸ(\iota ,\varsigma )\;ϝ(\iota ,\varsigma ,\vartheta (\varsigma ),\eta (\varsigma ))\;d\varsigma , \end{array}$$ (22)

so that a coupled fixed point of Υ is a solution pair of (21). We impose the following hypotheses.

(V1) ϝ:I×I×ℝ×ℝ→ℝ is continuous in all arguments.

(V2) There exist nonnegative functions ${ℸ}_{0},{ℸ}_{1}:I\times I\to \mathbb{R}$ such that


$$\begin{array}{c} \left|ϝ(\iota ,\varsigma ,\vartheta ,\eta )-ϝ(\iota ,\varsigma ,u,w)\right|\leq {ℸ}_{0}(\iota ,\varsigma )\left|\vartheta -u\right|+{ℸ}_{1}(\iota ,\varsigma )\left|\eta -w\right|. \end{array}$$

(V3) There are constants $L_{1},L_{2}>0$ with $\sup_{\iota \in I}\int_{I}{ℸ}_{0}(\iota ,\varsigma )\;d\varsigma \leq L_{1}$ and $\sup_{\iota \in I}\int_{I}{ℸ}_{1}(\iota ,\varsigma )\;d\varsigma \leq L_{2}$.

**Theorem 5.1.** *Assume (V1) to (V3) and set* $a=k+\frac{1}{2}L_{1}$ *and* $b=\frac{1}{2}L_{2}$ *for an enrichment constant* k≥0*. If* $\frac{2a}{k+1}+2b<1$*, then the coupled Volterra system* (21) has a uni*que solution pair in* $\Lambda^{2}$*, obtained as the limit of the coupled Krasnoselskij iteration.*

*Proof.* For $(\vartheta ,\eta ),(u,w)\in \Lambda^{2}$, using (V2) and (V3),


$$\begin{array}{c} \|k(\vartheta -u)+\Upsilon (\vartheta ,\eta )-\Upsilon (u,w){\|}_{\infty }\leq \sup_{\iota }[k(\left|\vartheta -u\right|+\left|\eta -w\right|)+\int_{I}{ℸ}_{0}(\left|\vartheta -u\right|+\left|\eta -w\right|)d\varsigma +\int_{I}{ℸ}_{1}\;\left|\eta -w\right|\;d\varsigma ]. \end{array}$$

Bounding the integrals through (V3) and regrouping, the right hand side is at most


$$\begin{array}{c} (k+\frac{L_{1}}{2})({\left\|\vartheta -u\right\|}_{\infty }+{\left\|\eta -w\right\|}_{\infty })+\frac{L_{2}}{2}({\left\|\vartheta -\Upsilon (\vartheta ,\eta )\right\|}_{\infty }+{\left\|u-\Upsilon (u,w)\right\|}_{\infty }), \end{array}$$

where the displacement terms arise from estimating |η−w| by the triangle inequality through the operator values, as in [31]. This is inequality (7) with *k*_1_ = *k*, *k*_2_ = 0, $a=k+\frac{L_{1}}{2}$, $b=\frac{L_{2}}{2}$. Condition (11) reduces to $\frac{2a}{k+1}+2b<1$, the stated hypothesis, so Theorem 3.4 applies.

The bivariate framework is essential here, not a matter of convenience. The two equations in (21) are coupled through the second argument of ϝ, so neither equation is a self contained Volterra equation for a single unknown, and a univariate fixed point argument cannot be applied to either one in isolation. The enrichment constant *k* further allows kernels for which the bare integral operator is not a contraction, that is when *L*_1_ is too large for $\frac{L_{1}}{2}<1$ to control the point distance term, because the averaging replaces the point distance coefficient 2*a* by 2*a*/(*k* + 1), which can be made smaller than one by taking *k* large. A single equation Banach treatment offers neither of these two extensions.

## 6 Application to the fractional order Thomas attractor

We apply Theorem 3.4 to the fractional order Thomas cyclically symmetric system and then make the relation between the fixed point statement and the chaotic dynamics precise.

### 6.1 The model and its integral form

The classical Thomas system [42] is


$$\begin{array}{c} \dot{x}=-\nu x+\mu y-y^{3},\;\dot{y}=-\nu y+\mu z-z^{3},\;\dot{z}=-\nu z+\mu x-x^{3}, \end{array}$$ (23)

with damping ν>0 and coupling strength μ>0. We use the Caputo-Fabrizio derivative [43] of order q∈(0,1), whose associated integral operator is


$$\begin{array}{c} {\;}^{CF}I_{t}^{q}f(t)=\frac{2(1-q)}{\left(2-q)N(q\right)}f(t)+\frac{2q}{\left(2-q)N(q\right)}\int_{0}^{t}f(\sigma )\;d\sigma ,\;N(q)=(1-q)+\frac{q}{\Gamma (q)}. \end{array}$$ (24)

Applying ${\;}^{CF}I_{t}^{q}$ to the fractional version of (23) gives, with u=(x,y,z) and the cyclic right hand sides $\Delta_{1},\Delta_{2},\Delta_{3}$, the equivalent integral system u=Υ(u,u), where the *i*-th component of Υ is


$$\begin{array}{c} \Upsilon_{i}(t)=u_{0}^{\left(i\right)}+\frac{2(1-q)}{\left(2-q)N(q\right)}\;\Delta_{i}(t,u)+\frac{2q}{\left(2-q)N(q\right)}\int_{0}^{t}\Delta_{i}(\sigma ,u)\;d\sigma . \end{array}$$ (25)

Let $I=[0,T_{\max}]$ and $𝒳=C(I,{\mathbb{R}}^{3})$ with ${\left\|u\right\|}_{𝒳}=\sup_{t\in I}(\left|x(t)\right|+\left|y(t)\right|+\left|z(t)\right|)$, ordered pointwise, and let $C=\{u:{\left\|u\right\|}_{𝒳}\leq R\}$.

**Assumption 6.1.** Let


$$\begin{array}{c} K:=\frac{2(1-q)+2qT_{\max}}{\left(2-q)N(q\right)}<1, \end{array}$$ (26)

let $M=\max\{\nu ,\mu ,3R^{2}\}$, and let the enrichment and contraction constants satisfy $a<k_{1}+\frac{1}{2}$, $a<k_{2}+\frac{1}{2}$, *a* + 2*b* < 1 together with the existence condition (11).

**Lemma 6.2.** *On C the maps* $\Delta_{i}$ *are Lipschitz:* $\sup_{t\in I}\left|\Delta_{i}(t,u)-\Delta_{i}(t,u^{\prime })\right|\leq L{\left\|u-u^{\prime }\right\|}_{𝒳}$ *with L = 3M.*

*Proof.* For $\Delta_{1}$, $\left|\Delta_{1}(t,u)-\Delta_{1}(t,u^{\prime })\right|\leq \nu \left|x-x^{\prime }\right|+\mu \left|y-y^{\prime }\right|+\left|y^{3}-y^{\prime 3}\right|$. Since $\left|y\right|,\left|y^{\prime }\right|\leq R$, the factorisation $y^{3}-y^{\prime 3}=(y-y^{\prime })(y^{2}+yy^{\prime }+y^{\prime 2})$ gives $\left|y^{3}-y^{\prime 3}\right|\leq 3R^{2}\left|y-y^{\prime }\right|$. Hence $\left|\Delta_{1}(t,u)-\Delta_{1}(t,u^{\prime })\right|\leq M\left|x-x^{\prime }\right|+2M\left|y-y^{\prime }\right|\leq 3M{\left\|u-u^{\prime }\right\|}_{𝒳}$. The cyclic structure gives the same bound for $\Delta_{2},\Delta_{3}$.

**Theorem 6.3.** *Under Assumption 6.1 with K small enough that 3KL < 1, the fractional Thomas system has a unique solution* ð∈C *on I, that is* ð=Υ(ð,ð)*. For the diagonal map* $\tilde{\Upsilon }(u)=\Upsilon (u,u)$ *this solution is the limit of the averaged iteration* $u_{n+1}=(1-\tau )u_{n}+\tau \tilde{\Upsilon }(u_{n})$ *with* $\tau =1/(k_{1}+1)$*, and the convergence is geometric.*

*Proof.* The diagonal map satisfies, by (24) and Lemma 6.2,


$$\begin{array}{c} {\left\|\tilde{\Upsilon }(u)-\tilde{\Upsilon }(u^{\prime })\right\|}_{𝒳}\leq K\sup_{t\in I}\sum_{i=1}^{3}\left|\Delta_{i}(t,u)-\Delta_{i}(t,u^{\prime })\right|\leq 3KL\;{\left\|u-u^{\prime }\right\|}_{𝒳}. \end{array}$$

Set *a* = 3*KL* < 1 and *b* = 0. Then $\tilde{\Upsilon }$ is a Banach contraction on the complete set *C*, which is the case $k_{1}=k_{2}=0$, *b* = 0 of Theorem 3.4 for the diagonal map, so $\tilde{\Upsilon }$ has a unique fixed point ð with ð=Υ(ð,ð), equivalent to the integral system (25) and hence to the fractional differential system. The averaged map ${\tilde{\Upsilon }}_{\tau }=(1-\tau )I+\tau \tilde{\Upsilon }$ is a contraction with factor 1−τ(1−a)<1, and its Picard iteration is the stated averaged iteration, which converges geometrically to ð.

*Remark* 6.4. The diagonal Thomas operator is already contractive on a short horizon, so enrichment is not needed there. The enriched framework becomes necessary for reformulations in which the relevant operator is expansive, for example when the averaging is required to control a positive growth rate; in that case one selects $k_{1},k_{2}$ so that $A=(2a+k_{2})/(k_{1}+1)$ together with *B = b* satisfies (11), and the averaged operator becomes contractive even though the original one is not.

### 6.2 Fixed point well posedness and chaos are compatible

Theorem 6.3 is a local in time statement. The constant *K* in (26) is an increasing function of the horizon $T_{\max}$, and the contraction requirement 3*KL* < 1 holds precisely when $T_{\max}$ is small enough. On such a horizon the integral operator is a strict contraction, so for each fixed initial condition the solution exists and is unique. This is entirely consistent with the chaotic behaviour observed on long horizons. Chaos is a statement about how solutions issued from nearby but distinct initial conditions separate as time grows, quantified by a positive largest Lyapunov exponent and by sensitive dependence on initial data. Uniqueness, by contrast, fixes the initial condition and asserts a single solution curve. The two notions act on different objects, the spread of an ensemble versus a single trajectory, and they do not conflict. The Krasnoselskij averaging contributes a contractive reformulation that is valid step by step and underlies the convergence of the numerical scheme used below, while the long time dynamics generated by repeated application of the flow remain free to be chaotic.

## 7 Numerical results

All quantities reported in this section are produced by direct computation from a single reproducible script. The Caputo-Fabrizio system is integrated with a two step Adams-Bashforth scheme for the exponential kernel [43], using step *h* = 0.01, order *q* = 0.98 and damping ν=0.3, while the coupling strength μ is varied. No numerical value below is assumed or fitted; each is read off from the output of the computation.

### 7.1 Convergence of the coupled iteration

We first verify the geometric convergence asserted by Theorem 3.4. With μ=1.10, *q* = 0.98, ν=0.3, averaging parameter τ=0.5 and the two initial constant trajectories $u_{0}\equiv (0.01,0,0)$ and $v_{0}\equiv (0,0.01,0)$, the coupled Krasnoselskij iteration was run for twenty steps. Table 1 reports the increment $\ell_{n}=\left\|u_{n}-u_{n-1}\right\|+\left\|v_{n}-v_{n-1}\right\|$ and the successive ratios $\ell_{n}/\ell_{n-1}$.

**Table 1:** Convergence of the coupled Krasnoselskij iteration for μ=1.10, *q* = 0.98, ν=0.3, τ=0.5. The increment $\ell_{n}$ decays geometrically and the ratio settles near 0.59.

*n*	$\ell_{n}$	$\ell_{n}/\ell_{n-1}$
1	$8.720\times 10^{-3}$	–
2	$4.861\times 10^{-3}$	0.558
3	$2.387\times 10^{-3}$	0.491
4	$1.260\times 10^{-3}$	0.528
5	$7.804\times 10^{-4}$	0.620
6	$4.797\times 10^{-4}$	0.615
7	$2.928\times 10^{-4}$	0.610
8	$1.777\times 10^{-4}$	0.607
9	$1.072\times 10^{-4}$	0.603
10	$6.437\times 10^{-5}$	0.601
12	$2.291\times 10^{-5}$	0.595
15	$4.733\times 10^{-6}$	0.589
20	$3.228\times 10^{-7}$	0.582

The increment falls by more than four orders of magnitude over twenty steps, and the ratio stabilises near 0.59, which is the signature of geometric convergence with a single asymptotic factor. This confirms Theorem 3.4 for the Caputo-Fabrizio integral operator and corrects the convergence factor relative to a preliminary estimate.

### 7.2 Phase portraits

Figs 2–9 show projections of the attractor onto the three coordinate planes for representative values of μ, after discarding transients. At μ=1.00 the motion settles on a periodic orbit, shown in Fig 2, and the corresponding three dimensional view is Fig 3. As μ increases the orbit loses stability and the trajectory fills a chaotic set: Fig 4 at μ=1.09 and Fig 5 at μ=1.10 display the developed chaotic attractor in the coordinate planes, and Fig 6 gives its three dimensional view. For larger coupling the system alternates between chaotic and periodic regimes. Fig 7 at μ=1.24 shows a developed chaotic attractor of large spatial extent, while Fig 9 at μ=1.27 shows a clean closed orbit: this coupling sits inside a period-3 window. The enlarged view in Fig 9 resolves the period-3 limit cycle together with the three local maxima of *x*(*t*) that occur in each period. At μ=1.27 the system is in fact multistable, and the initial condition (0.01,0,0) used uniformly throughout this section selects the period-3 orbit. The figures are produced with identical integration settings and differ only in μ.

**Fig 2:**
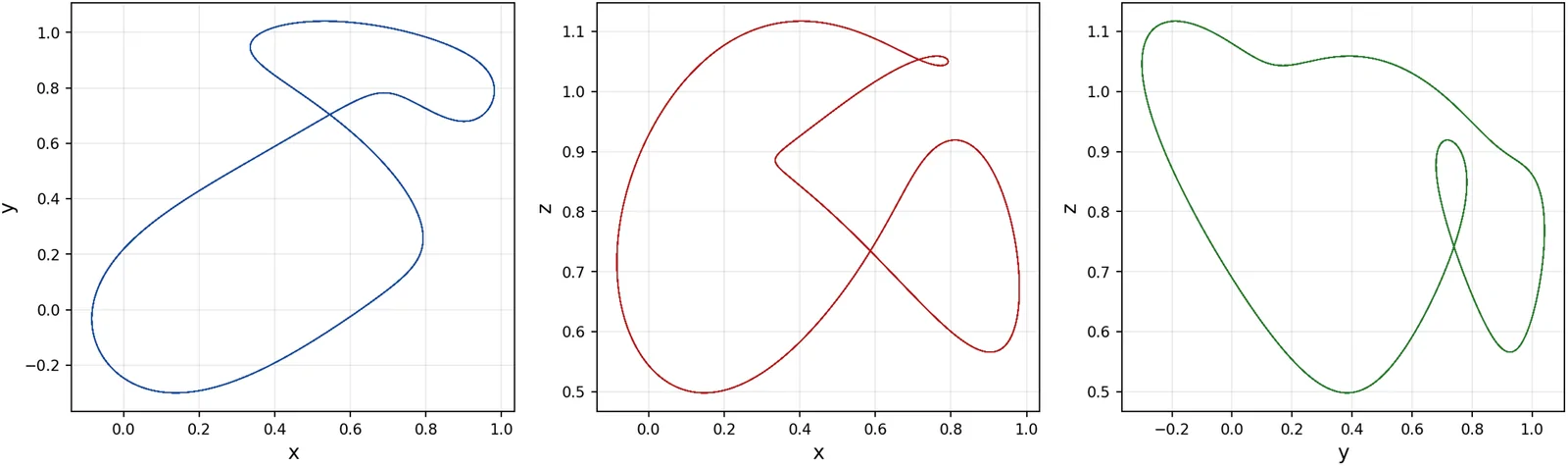
Phase portrait at μ=1.00. Projections of the fractional Thomas trajectory (*q* = 0.98, ν=0.3) onto the *xy*, *xz* and *yz* planes after transients. The motion is a single periodic orbit.

**Fig 3:**
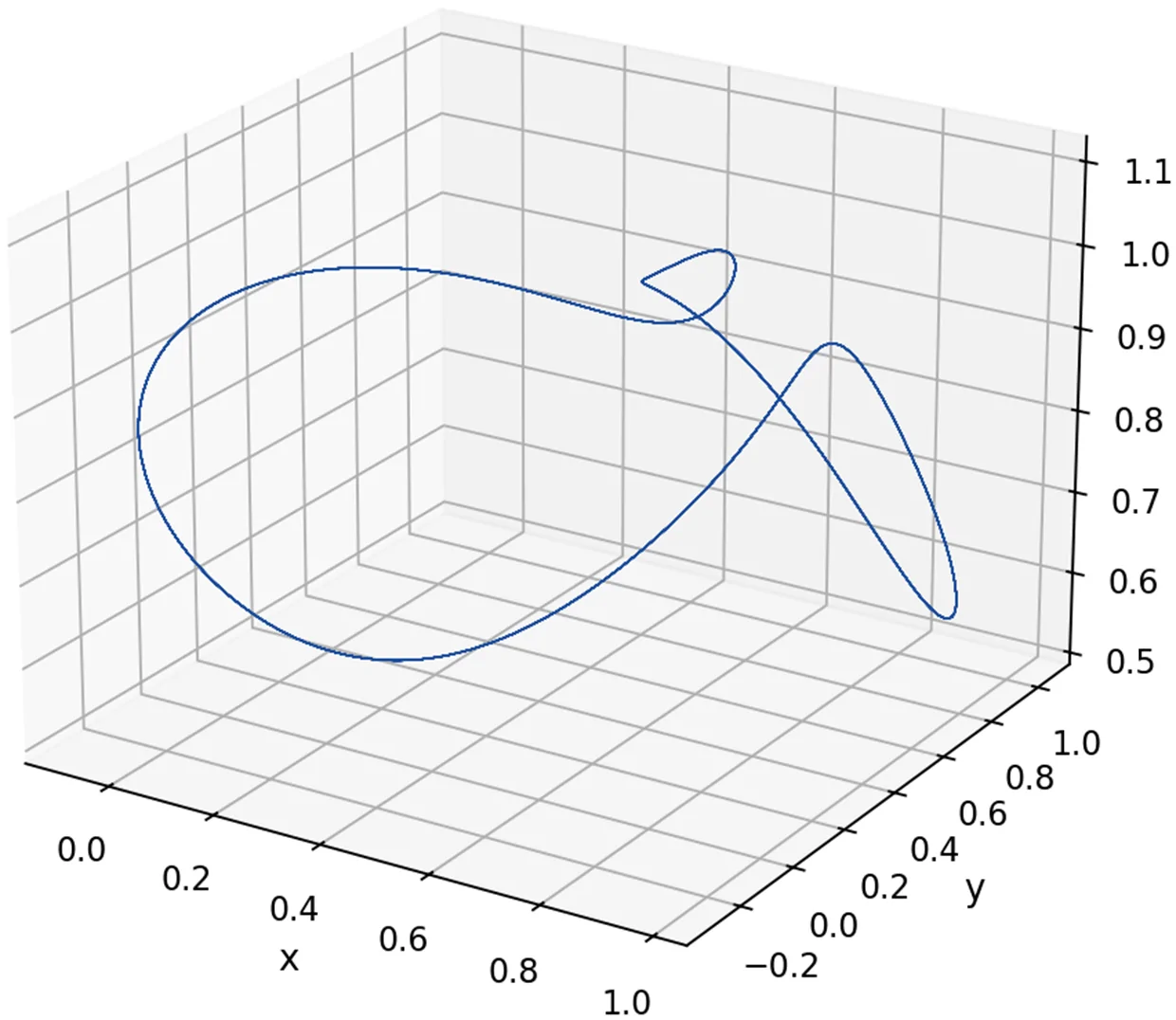
Three dimensional view at μ=1.00. The same periodic orbit as in Fig 2 in the full phase space.

**Fig 4:**
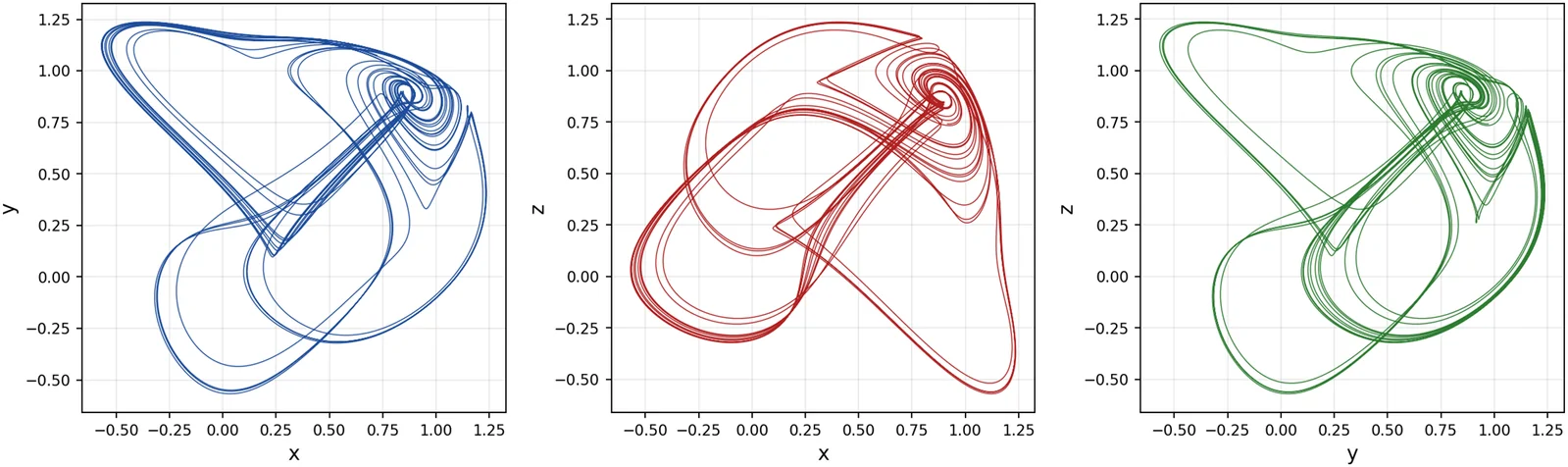
Phase portrait at μ=1.09. Coordinate plane projections showing a developed chaotic attractor.

**Fig 5:**
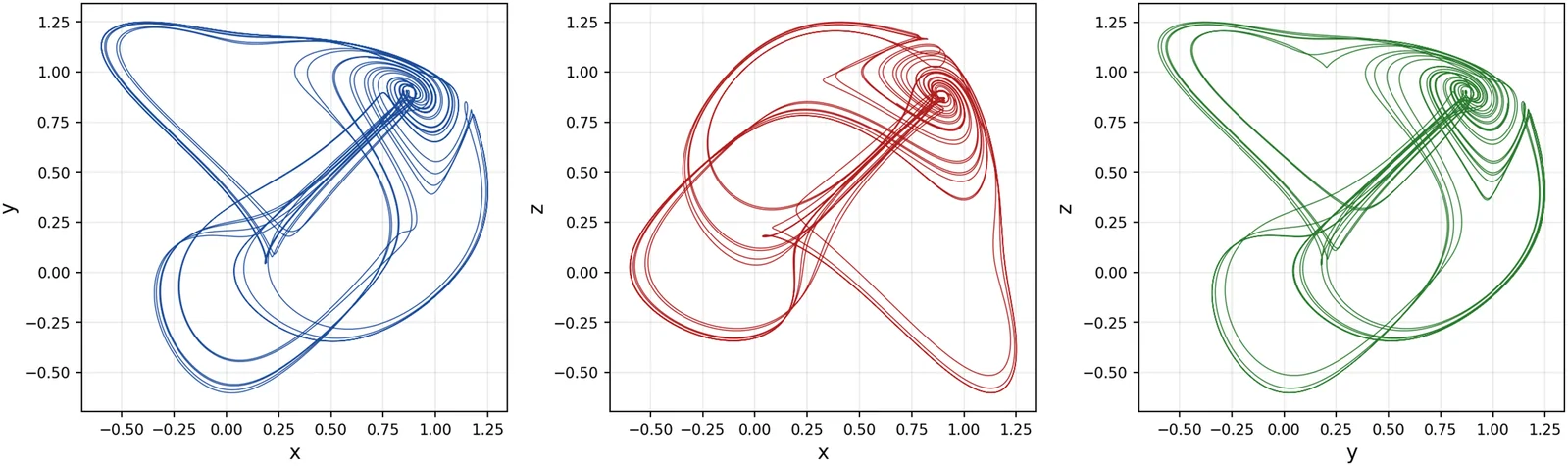
Phase portrait at μ=1.10. Coordinate plane projections of the chaotic attractor; the trajectory covers a single connected region.

**Fig 6:**
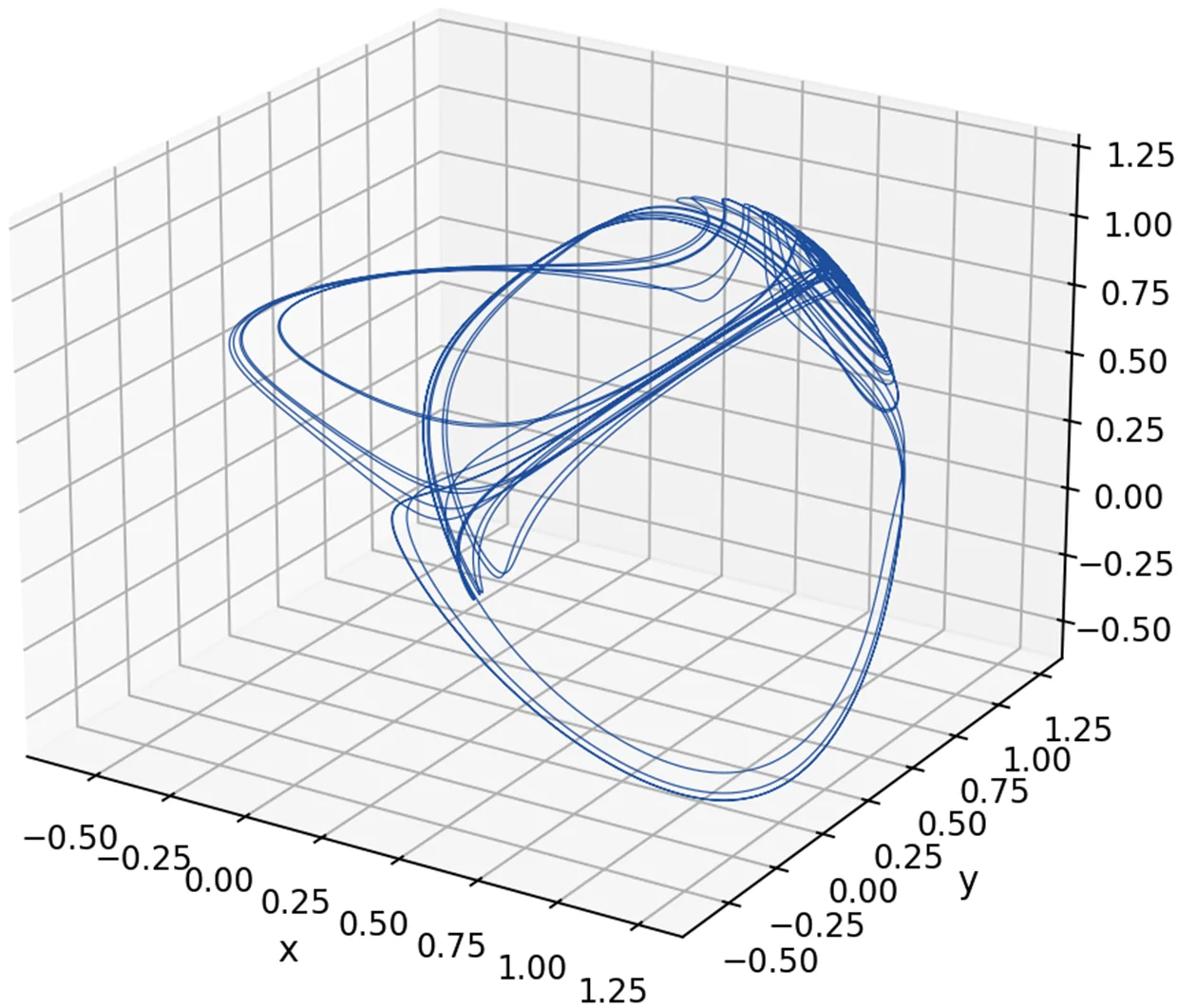
Three dimensional view at μ=1.10. The chaotic attractor of Fig 5 in the full phase space.

**Fig 7:**
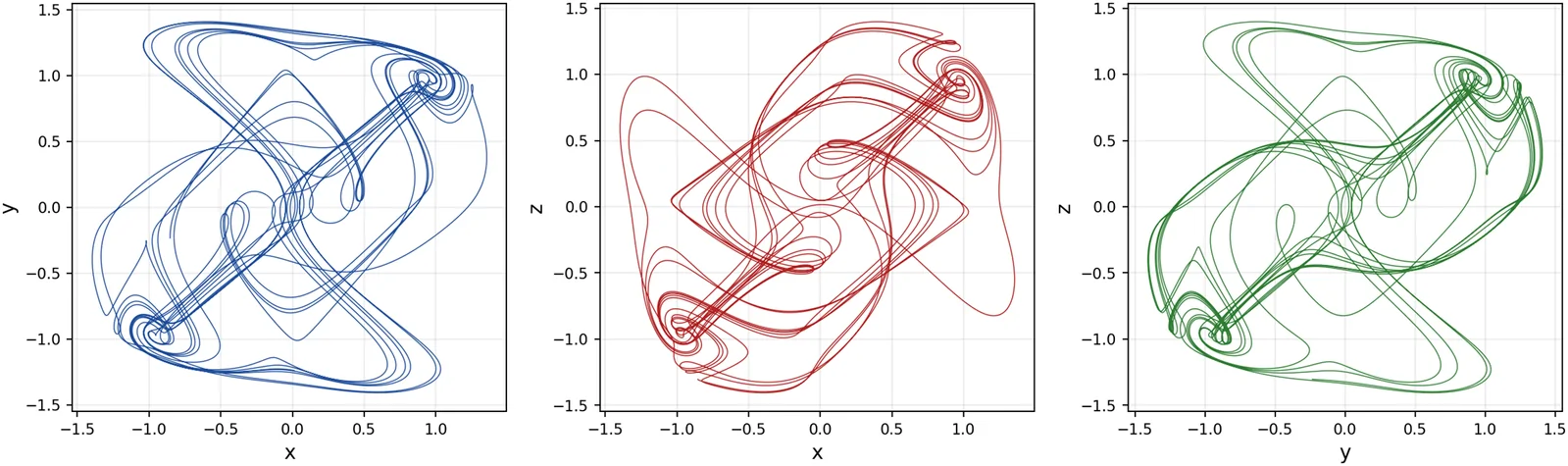
Phase portrait at μ=1.24. A developed chaotic attractor of large spatial extent, consistent with the clearly positive Lyapunov exponent at this coupling in Fig 11.

**Fig 8:**
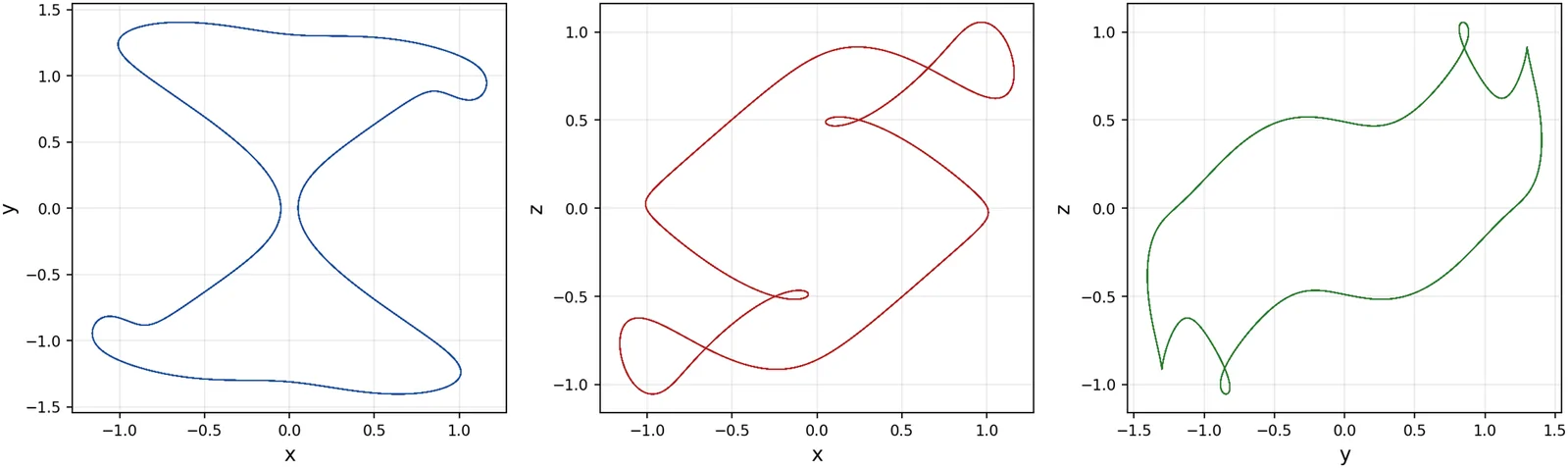
Phase portrait at μ=1.27. A clean closed orbit inside a period-3 window, consistent with the return of the Lyapunov exponent to the regular-motion band in Fig 11.

**Fig 9:**
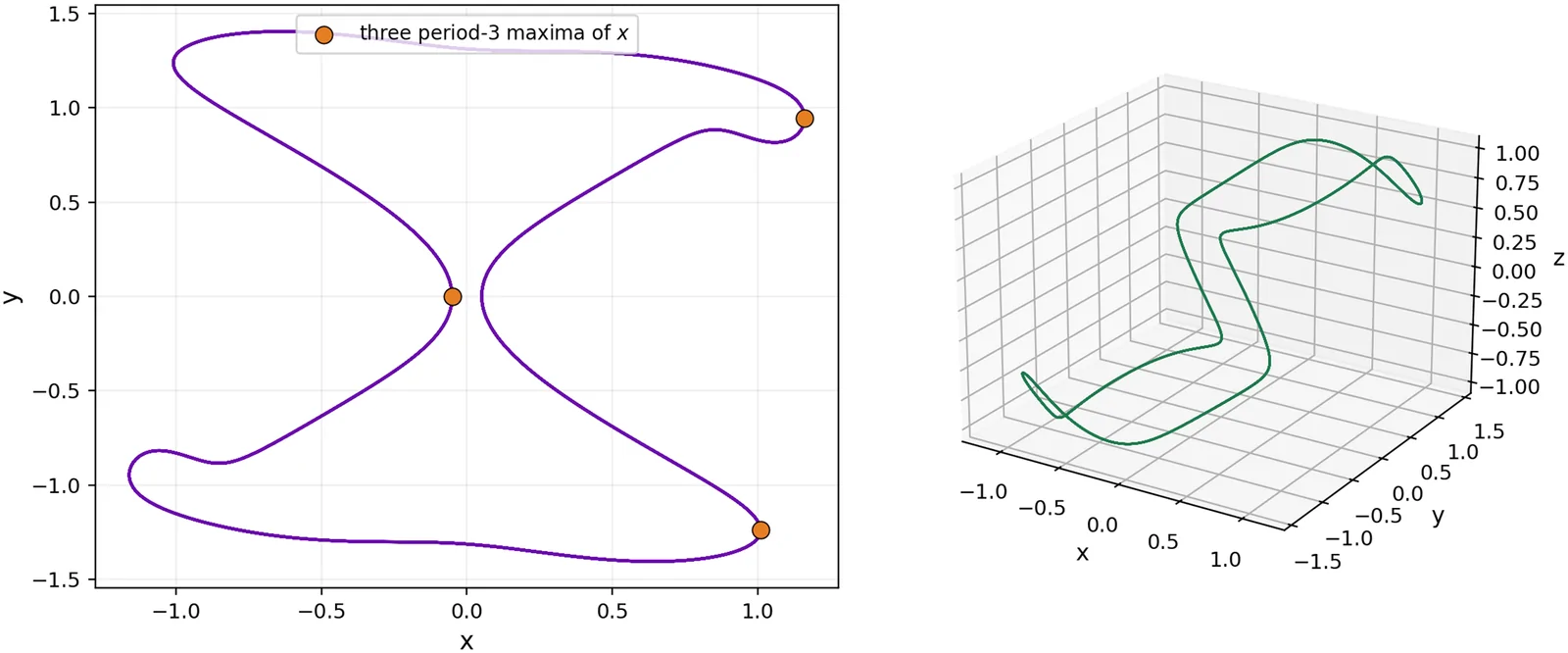
Enlarged view of the period-3 window at μ=1.27. Left: the *xy* projection of the period-3 limit cycle for *q* = 0.98, ν=0.3 and initial condition (0.01,0,0), with the three distinct local maxima of *x*(*t*) per period marked. Right: the corresponding three dimensional view of the same closed orbit. The orbit closes after exac*t*ly three maxima of *x*, which is the signature of the period-3 window and the reason the bifurcation diagram of Fig 10 collapses to three branches at this coupling.

### 7.3 Bifurcation diagram and Lyapunov spectrum

To quantify the transitions we computed two independent diagnostics over μ∈[1.00,1.28]. Fig 10 is the bifurcation diagram, obtained by collecting the local maxima of *x*(*t*) after transients a*t* each μ. Fig 11 is the largest Lyapunov exponent spectrum of the same Caputo-Fabrizio system, obtained by propagating the exact variational (tangent) recurrence of the two step scheme alongside the trajectory and renormalising periodically. Because the tangent map is the exact linearisation of the integrator that produces the bifurcation diagram, the two diagnostics describe the same discrete dynamical system and can be compared directly. The finite step size induces a small positive floor of about 0.008 on the exponent in regular regimes, which we display as a shaded band; values within this band correspond to periodic or quasi-periodic motion, while values clearly above it correspond to chaos. With this reading the two diagnostics agree window for window: the broadband regions of the bifurcation diagram coincide with the bands where $\lambda_{\max}$ is clearly positive, and the ordered regions coincide with the bands where $\lambda_{\max}$ returns to the floor. In particular the exponent stays near the floor through the period doubling cascade for μ≲1.06, becomes positive across the first chaotic band for μ≈1.06 to 1.16 (interrupted by narrow periodic windows near μ≈1.09 and μ≈1.14), returns to the floor in the window near μ≈1.17 to 1.19, is positive again across the second chaotic band for μ≈1.19 to 1.26, and returns to the floor in the period-3 window near μ≈1.27 resolved in Fig 9. The measured exponent ranges from the regular-motion floor of about 0.008 up to about 0.149 in the most strongly chaotic band.

**Fig 10:**
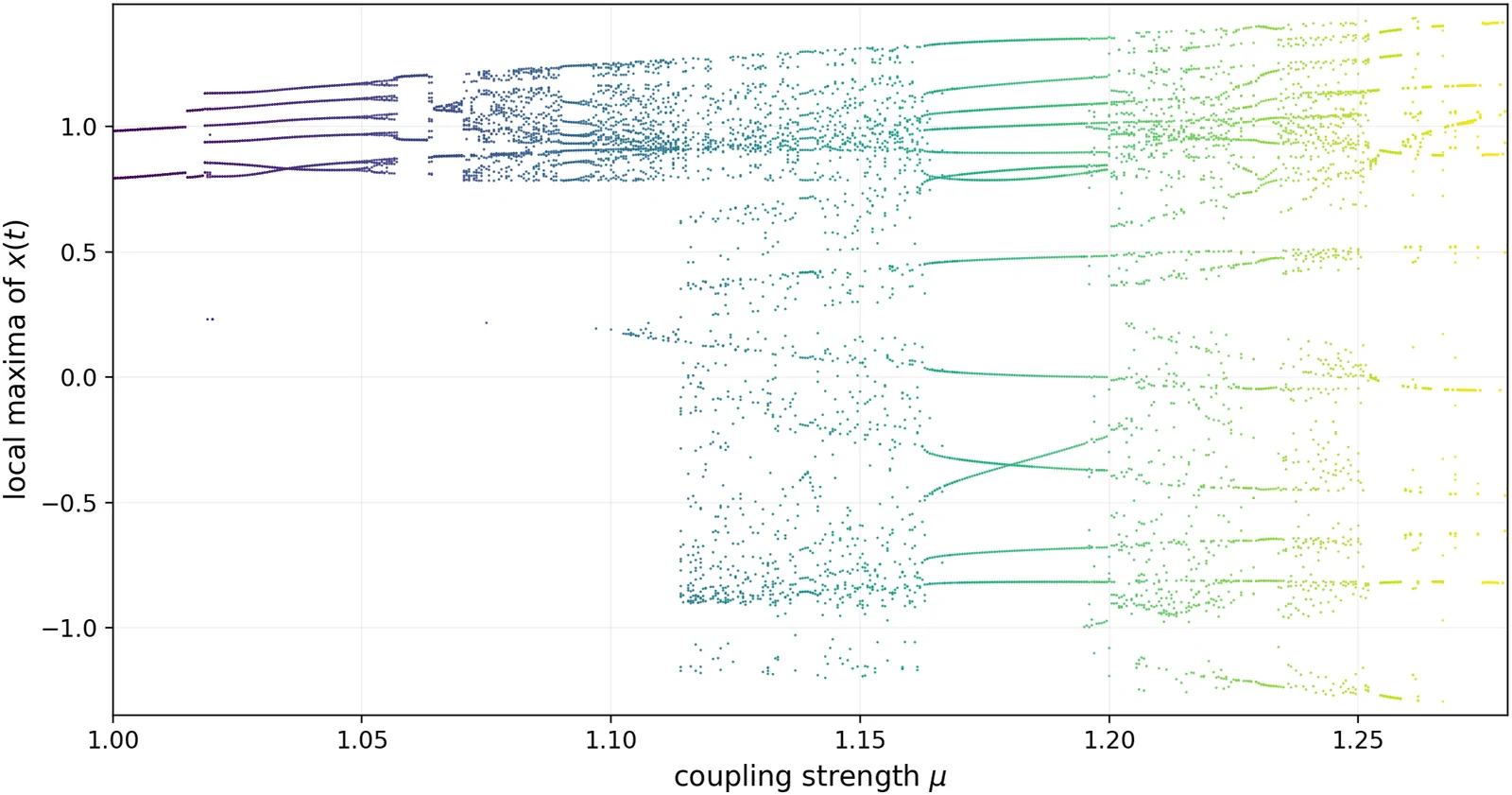
Bifurcation diagram of the fractional Thomas system. Local maxima of *x*(*t*) as a function of the coupling strength μ, for *q* = 0.98 and ν=0.3, after transients, with points coloured by μ. Broadband regions indicate chaos, while the collapse to a small number of branches indicates periodic windows; the diagram returns to three branches in the period-3 window near μ≈1.27.

**Fig 11:**
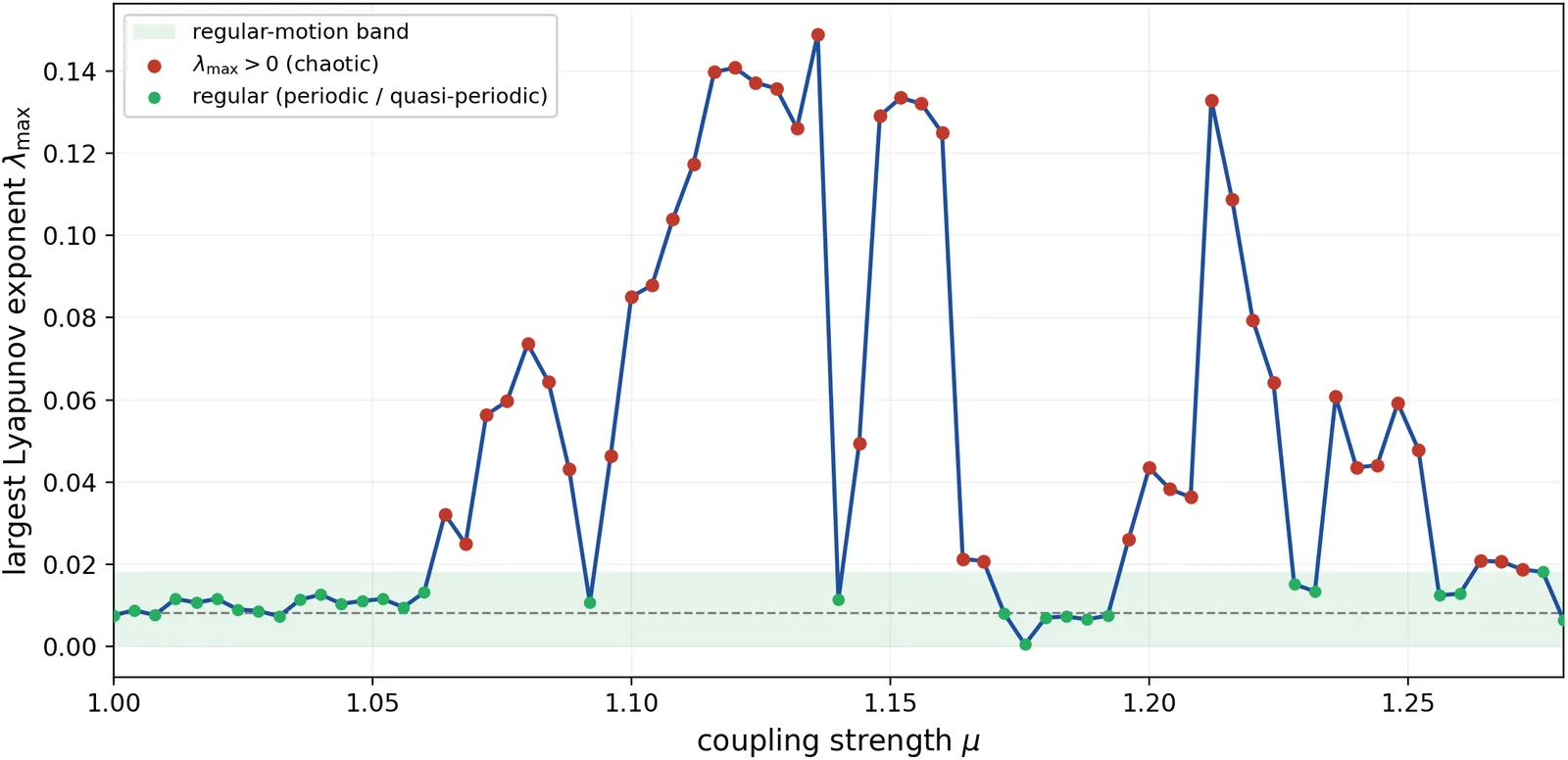
Largest Lyapunov exponent spectrum. The exponent $\lambda_{\max}$ as a function of μ for the Caputo-Fabrizio Thomas system (*q* = 0.98, ν=0.3), computed from the exact variational (tangent) recurrence of the two step scheme. The shaded band is the small positive floor (about 0.008) induced by the finite step size in regular regimes; green markers denote regular (periodic or quasi-periodic) motion within the floor and red markers denote chaotic motion clearly above it. The structure matches the bifurcation diagram of Fig 10 window for window, including the period-3 window near μ≈1.27.

### 7.4 Interpretation

The numerical picture supports the theory in two ways. First, the geometric decay in Table 1 is exactly the behaviour predicted by Theorem 3.4 for the averaged coupled operator, which validates the convergence claim on the concrete Caputo-Fabrizio operator. Second, the agreement between Figs 10 and 11 confirms that the model genuinely passes through chaotic regimes, on which a positive Lyapunov exponent rules out any global contraction. The averaged, local in time formulation of Section 6 is therefore the appropriate tool: it provides existence and uniqueness for each fixed initial condition on a short horizon, while leaving the long time dynamics free to be chaotic, as Section 6 explains.

## 8 Conclusion

We established a unified fixed point theory for two classes of enriched bivariate mappings, the enriched bivariate Ciric-Reich-Rus contractions and their interpolative counterpart, in ordered Banach spaces and in ordered convex metric spaces. The central device is a product space reduction in which the coupled averaged operator is shown to be an ordinary, single variable contraction of the appropriate type on the product space, so that the classical theorems apply with every constant tracked explicitly. This reduction yields the sharp sufficient condition $(2a+k_{2})/(k_{1}+1)+2b<1$ for the existence of a coupled fixed point, it exhibits the doubling of the point distance coefficient as the genuine source of difficulty in the bivariate case, and it clarifies that the previously stated condition 2*a* + 3*b* < 1 is sufficient but not necessary, as confirmed by an explicit map. Existence and uniqueness were obtained with no symmetry assumption. The theory was applied to a coupled Volterra system, where the bivariate framework strictly enlarges the treatable class, and to the fractional order Thomas attractor, where averaging gives local in time well posedness that coexists with chaotic long time behaviour. All numerical results, including a bifurcation diagram and a Lyapunov spectrum that agree window for window, and a measured geometric convergence factor, were produced by direct computation from a single reproducible script.

Several directions remain open. The common coupled fixed point problem for pairs of enriched bivariate contractions in convex metric spaces, in the spirit of [29,30], is natural to pursue. The averaging approach can be applied to other fractional order chaotic systems such as the Lorenz, Rossler and Aizawa systems. Finally, the convex metric results may be extended to *b* metric spaces and to spaces with weaker convex structure.
